# Validation of a new risk score system for non-variceal upper gastrointestinal bleeding

**DOI:** 10.1186/s12876-020-01346-4

**Published:** 2020-06-17

**Authors:** Min Seong Kim, Hee Seok Moon, In Sun Kwon, Jae Ho Park, Ju Seok Kim, Sun Hyung Kang, Jae Kyu Sung, Eaum Seok Lee, Seok Hyun Kim, Byung Seok Lee, Hyun Yong Jeong

**Affiliations:** 1 Division of Gastroenterology, Departmentof Internal Medicine, Chungnam National University Hospital, Chungnam National University School of Medicine, 282 Munhwa-ro, Jung-gu, Daejeon, 35015 Republic of Korea; 2 Clinical Trials Center, Chungnam National University Hospital, Daejeon, 34952 South Korea

**Keywords:** Gastrointestinal bleeding, Mortality, Risk assessment

## Abstract

**Background:**

Recently, a new international bleeding score was developed to predict 30-day hospital mortality in patients with upper gastrointestinal bleeding (UGIB). However, the efficacy of this newly developed scoring system has not been extensively investigated. We aimed to validate a new scoring system for predicting 30-day mortality in patients with non-variceal UGIB and determine whether a higher score is associated with re-bleeding, length of hospital stay, and endoscopic failure.

**Methods:**

A retrospective study was performed on 905 patients with acute non-variceal UGIB who were examined in our hospital between January 2013 and December 2017. Baseline characteristics, endoscopic findings, re-bleeding, admission, and mortality were reviewed. The 30-day mortality rate of the new international bleeding risk score was calculated using the receiver operating characteristic curves and compared to the pre-endoscopy Rockall score, AIMS65, Glasgow Blatchford score, and Progetto Nazionale Emorragia Digestiva score. To verify the variable for the 30-day mortality of the new scoring system, we performed multivariate logistic regression using our data and further analyzed the score items.

**Results:**

The new international bleeding scoring system showed higher receiver operating characteristic (ROC) curve values in predicting mortality (area under ROC curve 0.958; [95% confidence interval (CI)]), compared with such as AIMS65 (AUROC, 0.832; 95%CI, 0.806–0.856; *P* < 0.001), PNED (AUROC, 0.865; 95%CI, 0.841–0.886; *P* < 0.001), Pre-RS (AUROC, 0.802; 95%CI, 0.774–0.827; *P* < 0.001), and GBS (AUROC, 0.765; 95%CI, 0.736–0.793; *P* < 0.001). Multivariate analysis was performed using our data and showed that the 30-day mortality rate was related to multiple comorbidities, blood urea nitrogen, creatinine, albumin, syncope at first visit, and endoscopic failure within 24 h during the first admission. In addition, in the high-score group, relatively long hospital stay, re-bleeding, and endoscopic failure were observed.

**Conclusion:**

This is a preliminary report of a new bleeding score which may predict 30-day mortality better than the other scoring systems. High-risk patients could be screened using this new scoring system to predict 30-day mortality. The use of this scoring system seemed to improve the outcomes of non-variceal UGIB patients in this study, through proper management and intervention.

## Background

Acute upper gastrointestinal bleeding (AUGIB) is a common medical emergency, with an incidence of 84–160 cases per 100,000 individuals and a mortality rate of approximately 10% [1]. More than 70% of AUGIB cases are non-variceal UGIB (NVUGIB), and peptic ulcer bleeding is the main cause of NVUGIB. Despite the development of endoscopic therapies and pharmacological management, NVUGIB is still associated with considerable rates of mortality and morbidity, and high medical expenses [2–5].

The International Consensus Recommendations on the management of patients with NVUGIB recommend “early risk stratification” using validated prognostic scales [6]. A number of scoring systems have been devised to predict the outcome of patients with AUGIB, including the Rockall (RS) [7], pre-endoscopic Rockall (pre-RS) [8], Glasgow-Blatchford (GBS) [ 9], AIMS65 [10, 11], and ProgettoNazionaleEmorragiaDigestiva (PNED) [12] scores. There have been many studies on the outcomes of UGIB and continued study on which scoring systems can better predict mortality. However, limited research has been performed on a unified opinion or a new superior scoring system. Recently, based on an international multicenter study, a newly developed international bleeding score (INBS) was used to predict the 30-day hospital mortality in patients with AUGIB. In the previous study, INBS well predicted the 30-day mortality in UGIB patients compared to the other scoring systems [13]. In this study, we aimed to evaluate whether INBS is effective in predicting 30-day mortality in NVUGIB patients and to determine whether re-bleeding and endoscopic treatment failure were high in the patients with a relatively high INBS score.

## Methods

### Patients and data collection

We retrospectively included patients who visited the emergency room (ER) for AUGIB at the Chungnam National University Hospital in Daejeon, Korea from January 2013 to December 2017. Patients older than 18 years old who presented with NVUGIB symptoms (melena, hematemesis, coffee-ground vomiting, and/or hematochezia) were included in the study. The exclusion criteria for this study were as follows: patients with lower GI, obscure GI, and variceal bleeding; those with post procedural complications; and those who were lost to follow-up after 30 days from visiting the ER.

Data were collected for each patient by reviewing their medical chart. The data included demographic data, comorbidities, chief complaints at first visit, Glasgow Coma score at the time of the emergency room visit, diagnosis including cause of bleeding, presence of endoscopic treatment, outcomes of endoscopic treatment, 30-day in-hospital mortality, length of hospital stay, hemodynamics, and laboratory result. On the basis of these data, the pre-endoscopic Rockall, Glasgow Blatchford, AIMS65, and INBS were calculated on the time of admission. The INBS is measured based on age, comorbidity, and blood test and is weighted according to severity of each category (Table 1). Three endoscopists (HY Jeong, JK Sung, and HS Moon) with more than 15 years of experience in reviewing endoscopic findings using the picture archiving communication system (PACS) classified the findings as gastric ulcers (GUs), duodenal ulcers (DUs), Dieulafoy’s lesions, Mallory-Weiss syndrome, esophageal ulcers, angio-dysplasia, hemorrhagic gastritis, acute gastric mucosal lesion, and cancer-related bleeding. The data for each scoring system were entered by one researcher (MS Kim). Some verified scoring systems, for example, pre-endoscopy Rockall score, AIMS65, Glasgow Blatchford score, are automatically calculated in the electronical program in our hospital where this study was conducted. However, the new risk score system is not currently fully validated; thus, it was calculated manually. Moreover, to minimize errors and bias, the first author of this paper (MS Kim) managed it.

**Table 1:** International bleeding risk score for predicting 30-day mortality

Variable	Assigned score
Age	
60–74 years	1
≥ 75 years	2
Comorbidity	
Altered mental status	2
Liver cirrhosis	2
Disseminated malignancy	4
ASA score	
3	1
≥ 4	3
Blood tests	
Urea > 10 mmol/L	1
Albumin < 30 g/L	2
Creatinine	
100–150 μmol/L	1
> 150 μmol/L	2

### Definitions

The grading of overall health comorbidity was performed according to the American Society of Anesthesiologists (ASA) classification [14]. As in previous studies [13], we categorized the patients into the high-risk group or the low-to-moderate-risk group using a cut-off INBS score; the high-risk group included patients with an INBS score > 7, whereas the low-to-moderate-risk group included patients with an INBS score ≤ 7.

The primary outcome was in-hospital mortality at 30 days, defined as any death occurring during hospitalization. Secondary outcomes were assessed for recurrence of bleeding (re-bleeding), duration of hospital stay, and endoscopic hemostasis failure at the first visit in the high-risk group.

Re-bleeding was characterized as fresh hematemesis and/or melena associated with the development of shock (pulse > 100 beats/min, systolic blood pressure < 100 mmHg), or a reduction in the hemoglobin concentration > 2 g/dL for > 24 h. This also included cases of re-bleeding upon repeat endoscopy [15].

The hospitalization period was defined as the date from visiting the ER to the date of discharge. Endoscopic hemostasis failure at first visit was defined as instability at the time of first visit and inability to perform endoscopic hemostasis or vascular embolization after angiography or surgery due to endoscopic hemostasis failure.

### Statistical analyses

We compared each scores’ discriminative ability to predict 30-day mortality by calculating the area under the receiver operating characteristic curves (AUROCs) with 95% confidence intervals (CIs). The optimal cut-off score for predicting very high-risk patients in each scoring system was determined by the maximum Youden index, with a sensitivity and specificity of 95%. In addition, the performance of the scoring systems was assessed by calculating the sensitivity, specificity, percentage of patients classified as high risk, and the mortality rate among them.

Based on the risk factors previously mentioned in the scoring systems, univariate and multivariate logistic regression analyses were performed to evaluate the risk factors for predicting the 30-day mortality of NVUGIB patients visiting our hospital. A *P* < 0.05 was regarded as statistically significant. This was to ensure that the risk factors mentioned in the other scoring systems were actually a significant risk factor in our database. All statistical analyses were performed with the SPSS (version21.0, SPSS Inc., Chicago, IL, USA) and MedCalc (version18.11,MedCalc®, MedCalc Software, Belgium).

## Results

**Fig. 1:**
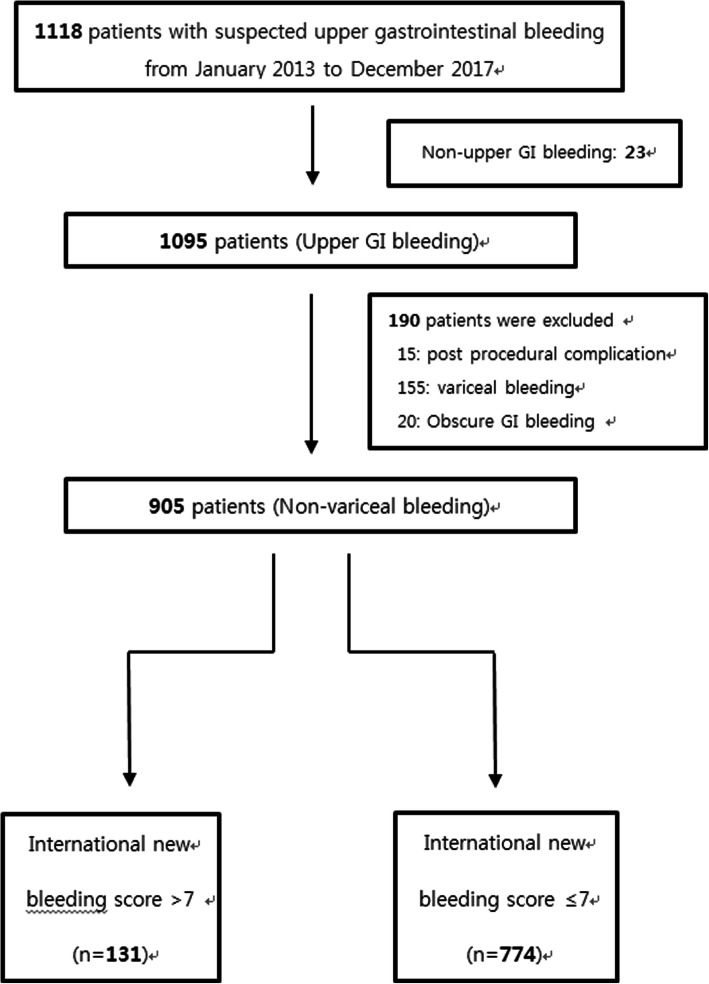
Flow chart of patients enrolled in this study. GI, gastrointestinal

Data of 1118 patients aged ≥18 years who visited the ER owing to UGIB for > 5 years were reviewed. We excluded 213 patients from the study for the following reasons: 23 patients had non-UGIB, 155 had variceal bleeding, 15 had postprocedure-associated bleeding, and 20 had obscure GI bleeding. In total, 905 patients were selected for evaluation. Of these, 131 and 774 patients were included in the high- and low-to-moderate-risk groups, respectively (Fig. 1).

### Patients’ characteristics

Of the 905 included patients, 674 (74.5%) were men and 231 (25.5%) were women, with a mean age of 65.4 ± 14.7 years. Upper GIT endoscopy was performed in all included patients. Seven hundred ten patients (78.4%) underwent endoscopy within 12 h during the admission in the emergency room, 160 patients (17.7%) underwent endoscopy within 12–24 h and 35 patients (3.9%) underwent endoscopy after 48 h. There were 35 patients who did not undergo endoscopy within 48 h of the first visit; most endoscopy procedures were performed after intensive care unit (ICU) hospitalization due to unstable vital signs. Among the total patients who underwent endoscopy, 735 received endoscopic hemostasis therapy; of these, patients underwent surgery (*n* = 11), trans-arterial embolization due to failure of the endoscopic treatment (*n* = 32), or both (*n* = 2). Among the patients who received endoscopic hemostasis, 128 had re-bleeding during the second endoscopy. Emergency endoscopic procedures were performed in most cases where patients had rebleeding during hospitalization and rebleeding after discharge. Thus, urgent endoscopic procedures within 12 h were performed in patients with rebleeding, and the mean average endoscopy timing in relation to rebleeding is 2.75 h.

Calculation of pre-RS, GBS, AIMS65, PNED, and INBS was possible in all cases. Table 2 shows the patients’ characteristics, chief complaint at first visit, treatment, and outcomes. The comorbidity scores were classified as ASA scores. The commonest endoscopic diagnosis was GU (67.8%), and the second commonest was DU (19.3%). There were 467 (51.6%) patients admitted to the hospital within 8 h. The average length of hospital stay for all patients was approximately 8.7 days. Among the 905 enrolled patients, only 31 patients (3.4%) were hospitalized for > 30 days A total of 44 patients (4.9%) died within 30 days in hospital. A total of 44 patients died in the hospital within 30 days. Of these, 14 had a direct association with non-variceal upper gastrointestinal bleeding, 12 died of heart failure, 11 of septic shock, 4 of liver failure, 2 of cerebral infarction, and 1 of cerebral hemorrhage. The mean INBS scores in the mortality and survivor groups were 8.38 ± 3.12 and 3.86 ± 2.56 (*P* value = 0.027), respectively.

**Table 2:** Baseline characteristics, treatment, and clinical outcomes of the study population

Patient related factor	All patients (***n*** = 905)
***Characteristics***	
Sex	
Male	674 (74.5%)
Female	231 (25.5%)
Age, years	65.4 ± 14.7
< 65	397 (43.9%)
≥65	508 (56.1%)
Alcohol	
Never	505 (55.8%)
Past	29 (3.1%)
Present	371 (41.0%)
Smoking	
Never	473 (52.3%)
Past	219 (24.2%)
Present	213 (23.5%)
ASA score 3	488 (53.9%)
ASA score 4	96 (10.6%)
Comorbidity	
Diabetes mellitus	251 (27.7%)
Hypertension	453 (50.1%)
Angina	81 (9.0%)
Cerebral infarction	156 (17.2%)
ARDS	28 (3.1%)
Disseminated malignancy	87 (9.6%)
Liver cirrhosis	67 (7.4%)
Sepsis	29 (3.2%)
DIC	26 (2.9%)
***Chief complaint at first visit***	
Melena	453 (50.1%)
Hematemesis	282 (31.2%)
Syncope	43 (4.8%)
Hematochezia	66 (7.3%)
Anemia	20 (2.2%)
Dizziness	28 (3.1%)
Others	13 (1.4%)
***Endoscopic findings of acute NVUGIB***	
Gastric ulcer	614 (67.8%)
Duodenal ulcer	175 (19.3%)
Gastroduodenal ulcer	11 (1.21%)
Esophageal ulcer	7 (0.77%)
Mallory-Weiss syndrome	47 (5.19%)
AGML	4 (0.44%)
Angiodysplasia	22 (2.43%)
Hemorrhagic gastritis	12 (1.32%)
Cancer-related bleeding	13 (1.76%)
Forrest classification (IA, IB, IIA)	607 (67.0%)
Forrest classification (IIB, IIC, III)	298 (32.9%)
***Outcomes***	
Admission before 8 h	467 (51.6%)
Admission day (mean)	8.779 day
Endoscopy failure at first admission	35 (3.9%)
Endoscopic hemostasis therapy	735 (81.2%)
Endoscopic hemostasis failure	52 (7.0%)
Re-bleeding at 2nd endoscopy	128 (14.1%)
All-cause mortality in hospital in 30 days	44 (4.9%)

Data are presented as mean ± SD, number (%), or mean (range)

*NVUGIB* Non-variceal upper gastrointestinal bleeding, *ASA* The American Society of Anesthesiology classification, *ARDS* Acute respiratory distress syndrome, *DIC* Disseminated intravascular coagulation, *AGML* Acute gastric mucosal Lesion

### Comparison of bleeding scores’ discriminative ability to predict the 30-day mortality

**Fig. 2:**
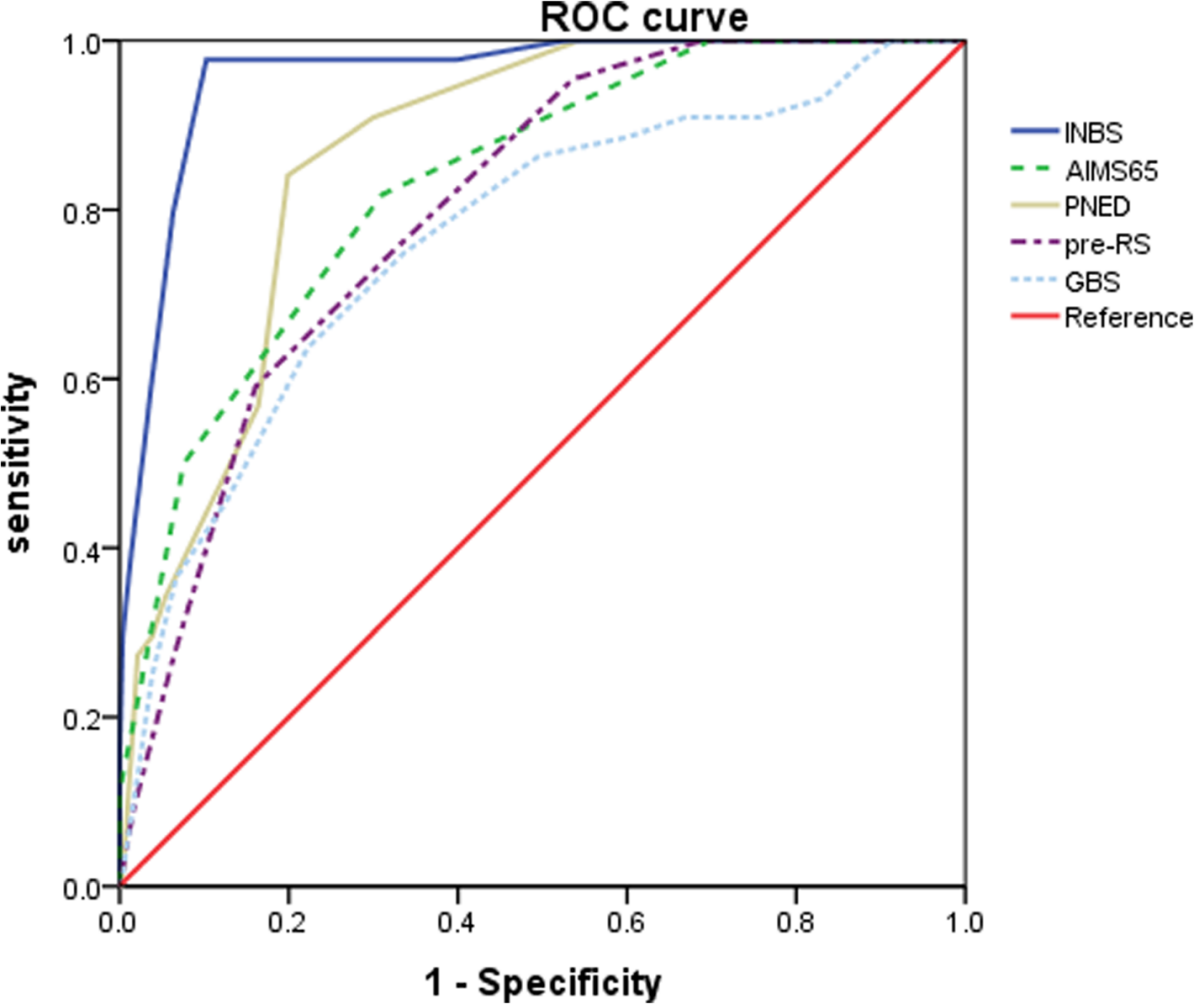
Comparison of scoring systems in the prediction of 30-day mortality (n = 905). AUROC, area under receiver operating characteristic curve [95% CI]; INBS, international new bleeding score; Pre-RS, pre-endoscopic Rockall score; GBS, Glasgow Blatchford score; PNED, Progetto Nazionale Emorragia Digestiva score

In this study, INBS had the highest discriminative ability (area under the receiver operating characteristic (AUROC) curve 0.958 [95% confidence interval (CI), 0.943–0.970]) in predicting mortality within 30 days compared with the AIMS65 (0.832; *P* < 0.001), PNED score (0.865; *P* < 0.001), Pre-RS (0.802; *P* < 0.001), and GBS (0.765; *P* < 0.001). The cut-off score was > 7 with a sensitivity of 97.73% and a specificity of 89.79% (Table 3 and Fig. 2). Table 4 shows the discriminative ability of the evaluated scoring systems to predict length of hospital stay, rebleeding, and endoscopic hemostasis failure.

**Table 3:** Predictive and discriminative abilities for identification of patients at high-risk of mortality in 30 days

System	Cut-off	High-risk n(%)	Sensitivity (%)	Specificity (%)	AUROC (95% CI)	Mortality n(%)	PPV (%)	NPV (%)
AIMS65	> 1	304 (33.6)	81.82	68.87	0.832 (0.806 to 0.856)	36 (11)	11.8	98.7
PNED	> 4	208 (23)	84.09	80.14	0.865 (0.841 to 0.886)	37 (17)	17.8	99
Pre-RS	> 4	165 (18.2)	59.09	83.86	0.802 (0.774 to 0.827)	26 (15)	15.8	97.6
GBS	> 12	219 (24)	63.64	77.82	0.765 (0.736 to 0.793)	28 (12)	12.8	97.7
INBS	> 7	131 (14.5)	97.73	89.79	0.958 (0.943 to 0.970)	43 (32)	32.8	99.9

*INBS* International bleeding risk score, *Pre-RS* Pre-endoscopic Rockall Score, *GBS* Glasgow Blatchford score, *PNED* Progetto Nazionale Emorragia Digestiva, *AUROC* Area under the receiver-operating characteristic curve, *CI* Confidence interval

**Table 4:** Discriminative ability of the evaluated scoring systems

Outcome by scoring system	AUROC (95% CI)
**Length of hospital stay**	
AIMS65	0.691 (0.660 to 0.721)
PNED	0.797 (0.769 to 0.823)
Pre-RS	0.697 (0.666 to 0.727)
GBS	0.641 (0.608 to 0.672)
INBS	0.710 (0.679 to 0.739)
**Re-bleeding rate**	
AIMS65	0.626 (0.593 to 0.657)
PNED	0.854 (0.829 to 0.876)
Pre-RS	0.643 (0.610 to 0.674)
GBS	0.621 (0.589 to 0.653)
INBS	0.636 (0.603 to 0.667)
**Endoscopic hemostasis failure**	
AIMS65	0.676 (0.645 to 0.707)
PNED	0.984 (0.973 to 0.991)
Pre-RS	0.686 (0.654 to 0.716)
GBS	0.688 (0.656 to 0.718)
INBS	0.710 (0.679 to 0.739)

*INBS* International bleeding risk score, *Pre-RS* Pre-endoscopic Rockall Score, *GBS* Glasgow Blatchford score, *PNED* Progetto Nazionale Emorragia Digestiva, *AUROC* Area under the receiver-operating characteristic curve, *CI* Confidence interval

The cut-off value was used to organize the patients into the high- and low-to-moderate-risk groups. A total of 131 patients (14.5%) were in the high-risk group; of these, 43 (32%) died within 30 days. The low-to-moderate-risk group comprised 774 patients (85.5%).

### Multivariate analysis for 30-day mortality

On the basis of the risk factors mentioned in the scoring system for UGIB patients, we performed logistic regression to identify predictors associated with mortality in patients who visited our hospital. In the univariate regression analysis, the variables that were meaningful were male sex, old age, smoking, ASA score of 4, hypertension, acute respiratory distress syndrome (ARDS), disseminated malignancy, liver cirrhosis, sepsis, disseminated intravascular coagulation, systolic blood pressure, heart rate, hemoglobin, platelet count, blood urea nitrogen (BUN), creatinine, international normalized ratio, syncope at first visit, endoscopic failure at first admission in 48 h, endoscopic hemostasis failure, and re-bleeding at the second endoscopy. Multivariate regression analysis was performed with the abovementioned variables that were significant in the univariate regression analysis (Table 5). Hypertension and systolic blood pressure could be duplicated, and only one was added. The multivariate analysis revealed that an ASA score of 4, ARDS, disseminated malignancy, creatinine, albumin, syncope at first visit, and endoscopic failure within 24 h during the first admission were associated with 30-day mortality.

**Table 5:** Univariate and multivariate analyses for 30-day mortality (*n* = 905)

Risk factor	Univariate analysis			Multivariate analysis		
	OR	95%CI	*P*value	OR	95%CI	*P*value
***Patient related factor***						
Sex (Male)	2.106	1.132–3.918	0.019	1.449	0.346–6.060	0.611
Age	1.031	1.007–1.056	0.012	1.017	0.967–1.069	0.507
Alcohol						
Never	1.000					
Present	1.548	0.842–2.844	0.159			
Smoking						
Never	1.000					
Present	0.442	0.228–0.857	0.016	0.383	0.106–1.385	0.143
ASA score 3	1.383	0.742–2.579	0.307			
ASA score 4	200.279	60.103–667.385	0.000	111.642	16.31–764.083	**0.000**
***Comorbidity***						
Diabetes mellitus	1.318	0.693–2.508	0.4			
Hypertension	2.481	1.281–4.806	0.007			
Angina	0.735	0.222–2.427	0.613			
Cerebral infarction	1.440	0.696–2.981	0.325			
ARDS	68.151	27.674–167.829	0.000	19.530	2.795–136.471	**0.003**
Disseminated malignancy	3.930	1.943–7.949	0.000	9.020	1.741–46.716	**0.009**
Liver cirrhosis	3.021	1.343–6.793	0.008	1.050	0.171–6.51	0.958
Sepsis	22.147	9.804–50.029	0.000	0.695	0.124–3.894	0.679
DIC	156.313	54.178–450.991	0.000	3.430	0.583–20.158	0.173
***Vital sign***						
SBP	0.968	0.954–0.982	0.000	1.026	1.000–1.052	0.052
HR	1.016	1.002–1.030	0.029	1.020	0.991–1.050	0.178
***Lab***						
Hb	0.803	0.706–0.914	0.001	0.901	0.679–1.194	0.468
PLT	0.993	0.989–0.998	0.002	0.999	0.993–1.005	0.707
BUN	2.356	1.277–4.346	0.006	0.334	0.083–1.349	0.124
Cr	1.012	1.003–1.020	0.006	1.434	1.031–1.996	**0.032**
Alb	0.099	0.053–0.186	0.000	0.182	0.045–0.726	**0.016**
INR	1.539	1.230–1.927	0.000	1.596	0.857–2.972	0.141
***Chief complaint at first visit***						
Syncope	19.449	9.488–39.865	0.000	5.985	1.294–27.687	**0.022**
***Admission and Endoscopy***						
Admission before 8 h	0.794	0.432–1.462	0.459			
Admissionday(≥ 8 days)	6.503	3.345–12.645	0.000	1.145	0.268–4.890	0.855
***Outcomes***						
Endoscopy failure at first admission	27.903	13.072–59.561	0.000	8.272	1.558–43.104	**0.012**
Endoscopy hemostasis failure	6.667	3.146–14.128	0.000	1.109	0.235–5.226	0.896
Re-bleeding at 2nd endoscopy	2.720	1.383–5.352	0.004	0.586	0.127–2.702	0.493

*ASA* The American Society of Anesthesiology classification, *ARDS* Acute respiratory distress syndrome, *DIC* Disseminated intravascular coagulation, *SBP* Systolic blood pressure, *HR* Heart rate, *Hb* Hemoglobin, *PLT* Platelet, *BUN* Blood urea nitrogen, *Cr* Creatinine, *Alb* Albumin, *INR* International normalized ration, *CI* Confidence interval

### Re-bleeding and length of hospital stay in the high-risk group

An INBS cut-off value > 7 was used to categorize patients into the high-score group (131 patients, 14.4%) and low-score group (774 patients, 85.5%). The high-score group had a relatively longer length of hospital stay and higher re-bleeding and endoscopic hemostasis failure rates than the low-score group (Table 6).

**Table 6:** Long-term hospital stay and occurrence of re-bleeding between high-risk group and low-to-moderate risk group in INBS

	High-risk group (*n* = 131)	Low-to-moderate risk group (*n* = 774)	*P* value^*^
Admission day(≥ 8 days)	86 (65%)	45 (5%)	< 0.001
Re-bleeding	36 (27%)	92 (11%)	< 0.001
Endoscopic hemostasis failure	23 (17%)	29 (3.7%)	< 0.001

*Chi-square

## Discussion

In this study, we evaluated whether INBS is effective in predicting 30-day mortality in NVUGIB patients and its utility in predicting rebleeding or hospitalization duration. INBS was superior to other pre-endoscopy risk scoring systems in predicting 30-day mortality.

AUGIB is a common medical emergency associated with high morbidity and 30-day mortality rates [1, 16]. More than 70% of AUGIB cases are NVUGIB, with GUs or DUs being the commonest. Mallory-Weiss syndrome, Dieulafoy’s ulcer, angiodysplasia, and cancer-related bleeding are also causes of NVUGIB [17]. While therapies such as Helicobacter pylori eradication therapy and proton pump inhibitors might be expected to reduce peptic ulcers and decreased the mortality rate of NVUGIB patients, studies show that the mortality rate is still high at 6 to 14% due to population aging and the use of anti-platelet drugs [18–21]. In these NVUGIB patients, timely hemostatic endoscopic procedures are important for survival improvement. For a successful treatment, assessing the hemodynamic status and appropriate risk measurement are necessary. Thus, classifying high-risk NVUGIB patients who have arrived at the ER using a highly efficient scoring system is important to help predict prognosis and direct appropriate treatment [7].

A variety of indicators (AIMS65 [10, 11], GBS [9], Pre-RS [8], PNED [12]) have been developed and evaluated to assess the risk of NVUGIB. Hyett et al. found that the AIMS65 score was superior to GBS in predicting death, but the GBS was better in predicting the need and the number of packed red blood cell transfusions [22]. In a cohort study of 424 participants, AIMS65 was also superior to GBS and Pre-RS in predicting hospital mortality, ICU admission, and loss of conscious [23]. In a European study, the usefulness of GBS, Pre-RS, and AIMS65 in patients with UGIB was assessed; however, there was no difference in mortality or re-bleeding frequency among the three scoring systems, and GBS was the best predictor of transfusion [24]. In another European study of 309 patients with UGIB, AIMS65, GBS, and Pre-RS were reported to be similar when predicting patient mortality; however, the need for endoscopic intervention was better predicted by AIMS65 and GBS [25, 26]. In a study on the utility of GBS and AIMS65 conducted in the United States and involving 165 patients with NVUGIB aged ≥65 years, GBS was superior to AIMS65 in predicting mortality [15]. The evaluation of the usefulness and predictability of the scoring system for assessing the prognosis of NVUGIB varies according to each study. The INBS^13^ scoring system that we have developed provides clear criteria for the 30-day mortality risk of patients with NVUGIB and has been shown to predict mortality better than the previous scoring systems. It is a predictive scoring system similar to the Pre-RS and PNED scores. However, INBS provides clear criteria for cirrhosis, differentiation of ASA scores according to hemodialysis, and blood tests to assess liver and renal functions. In contrast to previous scoring systems, the INBS included medical history including liver, renal and heart failure, in addition to the objective metabolic and biochemical indicators which we have described.

Our research suggests several strengths based on this new scoring system. First, this study validated INBS against the Pre-RS, GBS, AIMS65, and PNED risk stratification scores for predicting 30-day mortality. INBS was superior to other pre-endoscopy risk scores in predicting 30-day mortality. AIMS65, PNED, and Pre-RS showed higher mortality predictions with an AUROC > 0.8, but it had lower values compared to INBS. Secondly, variables considered as risk factors in each scoring system were confirmed in this study. In the multivariate analysis, an ASA score of 4, ARDS, disseminated malignancy, creatinine, albumin, syncope at first visit, and failure of endoscopic treatment within 24 h of the first visit were identified as risk factors for death in NVUGIB patients. The risk of death due to underlying disease factors, aggravation of liver and kidney diseases, and failure of endoscopic treatment at the first visit were confirmed. The significant death risk factors in this study were similar to the death risk factors already reflected in INBS. INBS has proposed criteria to identify liver and renal failures through liver cirrhosis, decreased albumin levels, and increased BUN and creatinine levels. The severity of the underlying disease was scored using the ASA classification. Thirdly, we classified the patients into the high- and low-to-moderate-risk groups based on the INBS cutoff value of > 7. The cut-off value was the same to that published in a previous study of ≥8 for the high-risk group [16]. In our study, we showed that the high INBS group had a relatively long hospital stay and high re-bleeding and endoscopic hemostasis failure rates. The cause of death from bleeding was one of the predictable outcomes if the patient was unable to tolerate hemostatic treatment due to a poor general condition or bleeding from cancer and massive bleeding from the large vessels. Therefore classification of patients using INBS could be used as a tool to increase the probability of successful hemostasis and to shorten the hospitalization period through rapid endoscopic treatment and intensive monitoring.

In Korea, tertiary medical care institutions properly and actively perform endoscopy in patients. Therefore it would be helpful for the doctors who perform such procedures to have a classification standard score to help determine the appropriate timing of endoscopy and to predict the prognosis of NVUGIB patients. Using INBS, the identification of patients with a high risk of death is possible, allowing targeted management and interventions that may improve outcome.

This study has some limitations. First, the present investigation was a retrospective, single-center study, which can be a confounding factor. Therefore, a larger sample size and a prospectively designed study are needed to confirm the effectiveness of INBS. Second, this is an observational study, which focused on a high-risk mortality factor and classified high-risk patients. However, the criteria for low-risk patients with low scores were not established, and neither the criteria for the need for endoscopic treatment nor those for outpatient follow-up were presented. Given the nature of the tertiary medical institutions, most patients visiting the ER with AUGIB are often referred from other medical institutions because of the severity of the disease. Thus, doctors often think that endoscopy should be performed or patients want to undergo endoscopy. Therefore, examining our study subjects is important to confirm the suitability of the score in selecting low-risk patients to receive outpatient treatment without endoscopy. In addition, the INBS scoring system is limited in its use as a criterion for selecting low-risk groups, because it is weighted towards identifying patients who are at risk of sudden aggravation or death from liver or kidney disease. Although this study has several limitations, we think that it has laid the foundation for larger, multi-center, prospective studies of the utility of the INBS scoring system. Specifically, such studies would be designed to investigate whether carrying out early endoscopy, and second-look endoscopy on the basis of the degree of risk identified by this new scoring system can improve patient outcomes.

## Conclusion

This is a preliminary report of a new bleeding score which may predict 30-day mortality better than the other scoring systems. High-risk patients could be screened using this new scoring system to predict 30-day mortality, longer hospital admission, re-bleeding, and endoscopic hemostasis failure. The use of this scoring system seemed to improve the outcomes of non-variceal UGIB patients in this study, through proper management and intervention.

## Data Availability

The datasets used and analyzed in the current study are available from the corresponding author on reasonable request.

## Abbreviations

AUGIB: Acute upper gastrointestinal bleeding
NVUGIB: Non-variceal upper gastrointestinal bleeding
INBS: International new bleeding score
RS: The Rockall score
Pre-RS: Pre-endoscopic Rockall score
GBS: Glasgow-Blatchford score
PNED: Progetto Nazionale Emorragia Digestiva
ER: Emergency room
ASA: American Society of Anesthesiologists
AUROC: The area under the receiver operating characteristic
CI: Confidence interval
GU: Gastric ulcer
DU: Duodenal ulcer
ARDS: Acute respiratory distress syndrome
BUN: Blood urea nitrogen

## References

[1] Kurien M, Lobo AJ (2015). Acute upper gastrointestinal bleeding. Clin Med (Lond).

[2] Abougergi MS, Travis AC, Saltzman JR (2015). The in-hospital mortality rate for upper GI hemorrhage has decreased over 2 decades in the United States: a nationwide analysis. Gastrointest Endosc.

[3] Leontiadis GI, Molloy-Bland M, Moayyedi P, Howden CW (2013). Effect of comorbidity on mortality in patients with peptic ulcer bleeding: systematic review and meta-analysis. Am J Gastroenterol.

[4] Sey MSL, Mohammed SB, Brahmania M, Singh S, Kahan BC, Jairath V (2019). Comparative outcomes in patients with ulcer- vs non-ulcer-related acute upper gastrointestinal bleeding in the United Kingdom: a nationwide cohort of 4474 patients. Aliment Pharmacol Ther.

[5] Gralnek IM, Dumonceau JM, Kuipers EJ, Lanas A, Sanders DS, Kurien M (2015). Diagnosis and management of nonvariceal upper gastrointestinal hemorrhage: European Society of Gastrointestinal Endoscopy (ESGE) guideline. Endoscopy..

[6] Barkun AN, Bardou M, Kuipers EJ, Sung J, Hunt RH, Martel M (2010). International consensus recommendations on the management of patients with nonvariceal upper gastrointestinal bleeding. Ann Intern Med.

[7] Rockall TA, Logan RF, Devlin HB, Northfield TC (1996). Risk assessment after acute upper gastrointestinal haemorrhage. Gut..

[8] Tham TC, James C, Kelly M (2006). Predicting outcome of acute non-variceal upper gastrointestinal haemorrhage without endoscopy using the clinical Rockall score. Postgrad Med J.

[9] Blatchford O, Murray WR, Blatchford M (2000). A risk score to predict need for treatment for upper-gastrointestinal haemorrhage. Lancet..

[10] Saltzman JR, Tabak YP, Hyett BH, Sun X, Travis AC, Johannes RS (2011). A simple risk score accurately predicts in-hospital mortality, length of stay, and cost in acute upper GI bleeding. Gastrointest Endosc.

[11] Nakamura S, Matsumoto T, Sugimori H, Esaki M, Kitazono T, Hashizume M (2014). Emergency endoscopy for acute gastrointestinal bleeding: prognostic value of endoscopic hemostasis and the AIMS65 score in Japanese patients. Dig Endosc.

[12] Marmo R, Koch M, Cipolletta L, Capurso L, Grossi E, Cestari R (2010). Predicting mortality in non-variceal upper gastrointestinal bleeders: validation of the Italian PNED score and prospective comparison with the Rockall score. Am J Gastroenterol.

[13] Laursen SB, Laine L, Dalton H, Murray IA, Schultz M, Ngu JH (2017). The international bleeding risk score: a new risk score that can accurately predict mortality in patients with upper GI-bleeding. Gastroenterology..

[14] Dobson G, Chong M, Chow L, Flexman A, Kurrek M, Laflamme C (2017). Guidelines to the practice of anesthesia - revised edition 2017. Can J Anaesth.

[15] Jung SH, Oh JH, Lee HY, Jeong JW, Go SE, You CR (2014). Is the AIMS65 score useful in predicting outcomes in peptic ulcer bleeding?. World J Gastroenterol.

[16] Robertson M, Majumdar A, Boyapati R, Chung W, Worland T, Terbah R (2016). Risk stratification in acute upper GI bleeding: comparison of the AIMS65 score with the Glasgow-Blatchford and Rockall scoring systems. Gastrointest Endosc.

[17] Holster IL, Kuipers EJ (2012). Management of acute nonvariceal upper gastrointestinal bleeding: current policies and future perspectives. World J Gastroenterol.

[18] Alexandrino G, Domingues TD, Carvalho R, Costa MN, Lourenço LC, Reis J (2019). Trends of endoscopy timing in patients with acute upper gastrointestinal bleeding. Clin Endosc.

[19] Nagasue T, Nakamura S, Kochi S, Kurahara K, Yaita H, Kawasaki K (2015). Time trends of the impact of *Helicobacter pylori* infection and non-steroidal anti-inflammatory drugs on peptic ulcer bleeding in Japanese patients. Digestion..

[20] Wuerth BA, Rockey DC (2018). Changing epidemiology of upper gastrointestinal hemorrhage in the last decade: a nationwide analysis. Dig Dis Sci.

[21] Li L, Geraghty OC, Mehta Z, Rothwell PM, Oxford Vascular Study (2017). Age-specific risks, severity, time course, and outcome of bleeding on long-term antiplatelet treatment after vascular events: a population-based cohort study. Lancet.

[22] Hyett BH, Abougergi MS, Charpentier JP, Kumar NL, Brozovic S, Claggett BL (2013). The AIMS65 score compared with the Glasgow-Blatchford score in predicting outcomes in upper GI bleeding. Gastrointest Endosc.

[23] Robertson M, Majumdar A, Boyapati R, Chung W, Worland T, Terban R (2016). Risk stratification in acute upper GI bleeding: comparison of the AIMS65 score with the Glasgow-Blatchford and rock-all scoring systems. Gastrointest Endosc.

[24] Budimir I, Gradiser M, Nikolic M, Barsic N, Ljubicic N, Kralj D (2016). Glasgow Blatchford, pre-endoscopic Rockall and AIMS65 scores show no difference in predicting rebleeding rate and mortality in variceal bleeding. Scand J Gastroentrerol.

[25] Martinez-Cara JG, Jimenez-Rosales R, Ubeda-Munoz M, de Hierro MJ, de Teresa J, Redondo-Cerezo E (2016). Comparison of AIMS65, Glasgow-Blatchford score, and Rockall score in a European series of patients with upper gastrointestinal bleeding: performance when predicting in-hospital and delayed mortality. United European Gastroenterol J.

[26] Tang Y, Shen J, Zhang F, Zhou X, Tang Z, You T (2018). Scoring systems used to predict mortality in patients with acute upper gastrointestinal bleeding in the ED. Am J Emerg Med.

